# Ammonia Toxicity in the Bighead Carp (*Aristichthys nobilis*): Hematology, Antioxidation, Immunity, Inflammation and Stress

**DOI:** 10.3390/toxics11030243

**Published:** 2023-03-05

**Authors:** Yuning Zou, Weixing Chen, Banghua Xia, Yifang Xiang, Zhentao Shen, Ying Han, Shuqun Xue

**Affiliations:** College of Animal Science and Technology, Northeast Agricultural University, Harbin 150030, China

**Keywords:** bighead carp, Ammonia stress, antioxidation, immunity, inflammation

## Abstract

Ammonia is one of the main environmental pollutants that affect the survival and growth of fish. The toxic effects on blood biochemistry, oxidative stress, immunity, and stress response of bighead carp (*Aristichthys nobilis*) under ammonia exposure were studied. Bighead carp were exposed to total ammonia nitrogen (TAN) concentrations of 0 mg/L, 3.955 mg/L, 7.91 mg/L, 11.865 mg/L, and 15.82 mg/L for 96 h. The results showed that ammonia exposure significantly reduced hemoglobin, hematocrit, red blood cell, white blood cell count, and platelet count and significantly increased the plasma calcium level of carp. Serum total protein, albumin, glucose, aspartate aminotransferase, and alanine aminotransferase changed significantly after ammonia exposure. Ammonia exposure can induce intracellular reactive oxygen species (ROS), and the gene expression of antioxidant enzymes (Mn-SOD, CAT, and GPx) increases at the initial stage of ammonia exposure, while MDA accumulates and antioxidant enzyme activity decreases after ammonia stress. Ammonia poisoning changes the gene expression of inflammatory cytokines; promotes the gene expression of inflammatory cytokines TNF-α, IL-6, IL-12, and IL-1β; and inhibits IL-10. Furthermore, ammonia exposure led to increases in stress indexes such as cortisol, blood glucose, adrenaline, and T3, and increases in heat shock protein 70 and heat shock protein 90 content and gene expression. Ammonia exposure caused oxidative stress, immunosuppression, inflammation, and a stress reaction in bighead carp.

## 1. Introduction

Ammonia nitrogen is the final product of the protein catabolism of fish, which is mainly excreted into the surrounding environment through the gills. Ammonia nitrogen in water exists in two forms, ionic ammonia (NH_4_^+^) and non-ionic ammonia (NH_3_). Among them, NH_3_ has good fat solubility, can freely passthrough biofilm and is more toxic to fish [1]. With the development of intensive aquaculture, a large number of residual bait and feces are accumulated and converted into ammonia nitrogen, which causes the ammonia content rise rapidly [2]. The exceedance of ammonia nitrogen has become a normal environmental problem [3]. Fish can usually rely on their own defense systems to improve ammonia poisoning when exposed to low ammonia, but exhibit significant symptoms of ammonia toxicity when the ammonia concentration is high [4,5]. Excessive accumulation of NH_3_ in fish can inhibit growth, disturb membrane potential and ion homeostasis, and lead to oxidative stress, tissue lesions, immunosuppression, and even death. These effects have been reported for fish such as grass carp (*Ctenopharyngodon idellus*) [6], pufferfish (*Takifugu obscurus*) [7], common carp (*Cyprinus carpio*) [8], Nile tilapia (*Oreochromis niloticus*) [9], Asian clam (*Corbicula fluminea*) [10], mudskipper (*Boleophthalmus pectinirostris*) [11], blunt snout bream (*Megalobrama amblycephala*) [12], and yellow catfish (*Pelteobagrus fulvidraco*) [13]. The impact of ammonia nitrogen stress on fish health has attracted more and more attention.

Ammonia exposure leads to an increase in reactive oxygen species (ROS) in the body, which damage DNA, proteins, and lipids, resulting in cell function damage [14]. The antioxidant defense system of cells can prevent or repair oxidative damage to different degrees. Recent studies have shown that the effects of ammonia on the antioxidant system were different according to fish species and ammonia exposure concentration, but the common factor was that high ammonia led to a decrease in antioxidant enzyme activity and caused oxidative stress [15]. When fish were under stress, the functions of the specific and non-specific immune defense systems were affected to varying degrees, resulting in increased susceptibility of the organism to various pathogens [16]. For example, the expression levels of tumor necrosis factor α (TNFα), and interleukin 1β (IL-1β) in young turbot (*Scophthalmus maximus*) were significantly up-regulated and the expression level of lysozyme (LZM) was significantly down-regulated when exposed to ammonia, which led to immunosuppression and an inflammatory reaction in turbot [17]. Ammonia nitrogen exposure induced elevated mRNA levels of TNFα, IL-6, and IL-12 in the liver of puffer fish, causing an inflammatory response [7]. Ammonia nitrogen, as an important pollutant in the aquatic environment, cannot be ignored for its harm to the antioxidant and immune systems of fish.

The bighead carp (*Aristichthys nobilis*) is a typical plankton-eating fish and an important link in the freshwater ecological food chain [18]. The study of the effects of ammonia exposure on the physiology of plankton-feeding fish has shown a very important ecological role as well as scientific value. In recent years, ammonia exposure has been a major environmental problem in bighead carp culture, which affects the yield and quality of this breed. To date, the research on the physiological effects of ammonia exposure on bighead carp has been limited. However, there are relevant studies indicating that chronic exposure of ammonia at lower concentrations can result in some degree of impairment of antioxidative function, and significant histological changes were found in livers and gills at high concentrations of ammonia [19,20]. This study comprehensively evaluated and analyzed the effects of ammonia nitrogen on liver and serum biochemistry, immunity, antioxidation, and stress of bighead carp, and provided new insights for the aquatic toxicology mechanism of plankton-eating fish induced by ammonia. To our knowledge, this study is the first detailed study on the physiological response of bighead carp under ammonia exposure.

## 2. Materials and Methods

### 2.1. Experimental Management

Bighead carp were obtained from the Northeastern Agricultural University (NAU) farming base. A total of 1500 3-month-old bighead carp with average body weight (153.66 ± 0.27 g) and average body length (9.91 ± 0.12 cm) were kept in 30 water culture system tanks (200 L) for 14 d before the start of the experiment. The natural light cycle was maintained, and water conditions were dissolved oxygen (DO) concentration ≥ 6 mg/L, average ammonia concentration in the fish farm facilities < 0.2 mg/L, total ammonia nitrogen (TAN) concentration ≤ 0.05 mg/L, nitrite < 0.5 mg/L, pH 7.5 ± 0.2, and water temperature 23.0 ± 0.5 °C with salinity < 1 mg/L.

A standard stock solution of 1 g/L NH_4_Cl was prepared (Reagent No. 75-09-2, Sinopharm Chemical Reagent Co., Ltd., Shanghai, China). The concentration of TAN, the pH, and salinity were tested every 12 h and 1/3 of the water was changed every 24 h. NH_4_Cl standard stock solution was added to maintain the TAN concentration, and NaOH and HCl standard stock solution was were to maintain the pH. NaCl standard stock solution was added to maintain the salinity. Standard stock solutions of 1 g/L NaCl (Reagent No. 7647-14-5, Sinopharm Chemical Reagent Co., Ltd., Shanghai, China), 1 g/L NaOH (Reagent No. 1310-73-2, Sinopharm Chemical Reagent Co., Ltd., Shanghai, China), and 1 g/L HCl (Reagent No. 7647-01-0, Sinopharm Chemical Reagent Co., Ltd., Shanghai, China) were prepared. Experimental fish were fed twice a day. The actual non-ionic ammonia (NH_3_) concentration in the water was calculated according to the method described by Emerson et al. [21].

No observed adverse effect level (NOAEL) = 0.196h-LC50.

### 2.2. Determination of Semi-Lethal Concentrations (LC50)

A total of 10 groups were set up with different TANs of 0 (control), 5, 10, 15, 20, 25, 30, 35, 40 and 50 mg/L. Five parallel groups were set up for each concentration, and 20 fish were placed in each parallel group. The death number of each concentration group was recorded at 0 h, 6 h, 12 h, 24 h, 48 h, 72 h, and 96 h, and the semi-lethal concentrations were calculated using SPSS 22.0. Calculation and regression equation chi-square (χ^2^) were used to test different concentrations of observed and expected values from 001 to 0∙99 mortality concentrations (including 0∙50, LC50). From the results, the 96 h LC50 probability unit model equation was obtained as PROBIT (*p*) = −3.440 + 2.869x, and the Pearson model goodness of fit test χ^2^ = 11.085, *p* = 0∙135 indicated a good model fit. This led to LC50 (Prob = 0∙50). The experiment was conducted for 96 h and the experimental fish were fasted.

### 2.3. Ammonia Exposure Experiment

Based on the 96-LC50 results, one control group and four experimental groups were set up at 0%, 25%, 50%, 75%, and 100% LC50 concentrations, which were labeled CTRL, AN1, AN2, AN3, and AN4 groups, with TAN concentrations of 0 mg/L, 3.96 mg/L, 7.91 mg/L, 11.87 mg/L, and 15.82 mg/L. Each concentration group was equipped with 5 parallel groups, each with 50 parallel fish. The experiment was carried out for 96 h and the fish were fasted. The experimental conditions are shown in Table 1.

### 2.4. Sampling

Ten fish were randomly sampled from each group at 0 h, 12 h, 24 h, 48 h, and 96 h of ammonia exposure, submerged in 5 ppm MS-222 (Sigma, St. Louis, MA, USA), and anesthetized for 30 s. Blood was collected for hematological parameter analysis. Serum and liver samples were quickly frozen in liquid nitrogen and stored at −80 °C for enzyme activity, protein content, and gene expression analysis.

### 2.5. Hematology

Whole blood was measured using an automated hematology analyzer (XE-2100, Sysmex, Kobe, Japan) for white blood cell (WBC), red blood cell (RBC), hemoglobin (HGB), platelet (Platelet, PLT) blood and hematocrit (Hematocrit, Ht). We added the sample directly to obtain the measurement results.

Whole blood centrifugal separation of serum was performed (4 °C; 10,000 r/min; 5 min). Serum total protein (TP, A045-2-2), albumin (ALB, A028-1-1), total cholesterol (CHOL, A111-1-1), triglycerides (TG, A110-1-1), glucose (GLU, F006-1-1), glutamic–pyruvic transaminase (ALT, C009-2-1), and glutamic oxalacetic transaminase (AST, C010-1-1) activities were measured using an automated biochemical analyzer (PUZS-300, Shanghai, China), and the assay kits were purchased from Nanjing Jiancheng Institute of Biological Engineering.

### 2.6. Cytokines and Immunological Indicators

Serum LYZ (A050-1-1), complement C3 (H186-1-2), AKP (A059-1-1), and serum cytokines, such as IL-1β (H002-1-2), IL-6 (H007-1-1), IL-8 (H008-1-1), IFN-γ (H025-1-2), TNF-α (H052-1-2), and IL-10 (H009-1-2) were tested according to the method specified in the kit, which was purchased from Nanjing Chengjian Institute of Biological Engineering.

### 2.7. Antioxidant Reaction

The liver samples were homogenized with phosphate buffer (4 °C, pH 7.4), and the supernatant was separated by centrifuge (4 °C, 2000 r/min, 15 min); the phosphate buffer was from Nanjing Jiancheng Institute of Biological Engineering. The activities of SOD (A001-3-2, WST-1 method), CAT (A007-1-1, Visible light method), and GPx (H545-1-2), and the contents of T-AOC (A015-1-2, colorimetry method), MDA (A003-1-2, TBA meathod), and ROS (E004-1-1, ChemiLuminescence method) were determined using biochemical kits from Nanjing Jiancheng Institute of Biological Engineering.

### 2.8. Stress Response

Plasma cortisol, 3′-triiodothyronine (T3), and adrenaline were measured by automatic chemiluminescence immunoassay (MAGLUMI 1000) and an ELISA quantitative kit. The content of HSP70 and HSP90 in the liver were measured by ELISA quantitative kit (Shanghai Enzyme-Linked Biology). The antibodies were pre-coated in the enzyme well included with the kit.

### 2.9. Gene Expression

Total liver tissue RNA was extracted by the Trizol method, and the integrity of the extracted RNA was assessed by 1.0% agar gel electrophoresis. The cDNA was synthesized according to the instructions from BioRT Master HiSensi cDNA First Strand Synthesis kit (BIOER, Hangzhou, China). The PCR system was 95 °C for 3 min; 95 °C for 10 s, 57–61 °C; and 95 °C for 5 s.The *β-actin* was used as an internal reference gene for the experiments, and the 2-^∆∆^Ct method was used to calculate the relative expression (R). The *β-actin* is present in all eukaryotic cells and is highly conserved, so it is often widely used as a reference gene. In this study, the β-actin gene of silver carp was used as a reference gene and primers were designed using Primer Premier 5 software based on the *HSP70 HSP90* mRNA sequence of NCBI published Danio rerio. All primers were ordered from Comate Bioscience Co., Ltd. (Tianjin, China), and the primers are shown in Table 2. The target gene and *β-actin* gene cDNA were quantified for each tissue sample using a Roche LightCycler 480 II instrument, referring to the BioEasy Master Mix (SYBR Green, High ROX) Real Time PCR Kit (BIOER, Hangzhou, China) instructions. All samples were amplified three times.

### 2.10. Statistical Analysis

The data were analyzed by the normal distribution and the homogeneity of variances and one-way analysis of variance (one-way ANOVA) using SPSS 22.0 (Chicago, IL, USA), followed by Duncan for multiple comparisons, and histograms were plotted by GraphPadPrism8.

### 2.11. Ethical Approval

This experiment strictly conformed to the recommendations of *The Management and Use Guide of Experimental Animals* of Northeast Agricultural University. This procedure was approved by the Animal Experiment Ethics Committee of Northeast Agricultural University under the following ethical approval identification number: NEAUEC20220208.

## 3. Results

### 3.1. Semi-Lethal Concentrations of Bighead Carp Exposed to Ammonia

According to SPSS analysis, the semi-lethal TAN concentrations (LC50) for bighead carp were 26.05 mg/L, 21.01 mg/L, 17.06 mg/L, and 15.82 mg/L for 24 h, 48 h, 72 h, and 96 h of ammonia exposure, respectively, and the NOAEL concentration was 1.58 mg/L. The concentrations of semi-lethal NH_3_ were 1.41 mg/L, 1.13 mg/L, 0.92 mg/L, and 0.85 mg/L for 24 h, 48 h, 72 h, and 96 h of ammonia exposure, respectively, and the NOAEL concentration was 0.09 mg/L, as shown in Table 3.

### 3.2. Effects of Ammonia Exposure on Blood Physiology

**Figure 1:**
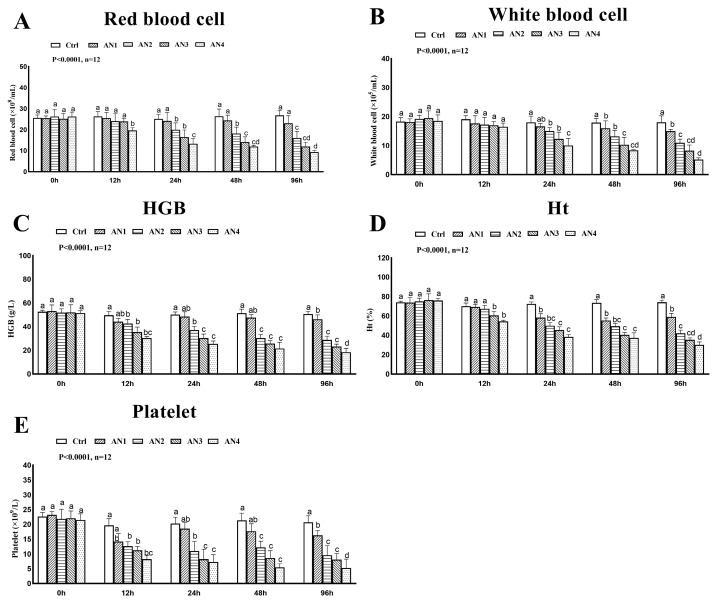
Changes of hematological parameters of bighead carp exposed to ammonia. (**A**) Red blood cell (RBC), (**B**) white blood cell (WBC), (**C**) hemoglobin (HGB), (**D**) hematocrit (Ht), (**E**) platelet (PLT). Note: Values are expressed as mean ± standard error (SE) from triplicate groups. Bars with different letters (a, b, c, d) differ significantly from those of other groups (*p* < 0.05).

The hematological parameters of bighead carp under ammonia exposure are shown in Figure 1. The RBC of the AN1 group was not significantly different from the control group. After exposure to ammonia for 24 h, 48 h and 96 h, the RBC of the AN2, AN3, and AN4 groups decreased significantly, as shown in Figure 1A. The WBC of each group was not significantly different from the control group at 12 h of ammonia exposure. The WBC of the AN2, AN3, and AN4 groups decreased significantly at 24 h, 48 h, and 96 h of ammonia exposure, Figure 1B. At 24 h, 48 h, and 96 h of ammonia exposure, the HGB of the AN2, AN3, and AN4 groups decreased significantly, that of AN1 group decreased at 96 h, Figure 1C. After 24 h, 48 h, and 96 h of ammonia exposure, HT in the AN1, AN2, AN3, and AN4 groups decreased significantly, Figure 1D. At 12 h, 24 h, 48 h, and 96 h of ammonia exposure, PLT in the AN2, AN3, and AN4 groups significantly decreased. After ammonia exposure of 96 h, PLT in the AN1 group significantly decreased, Figure 1E.

### 3.3. Effect of Ammonia Exposure on Plasma Composition

**Figure 2:**
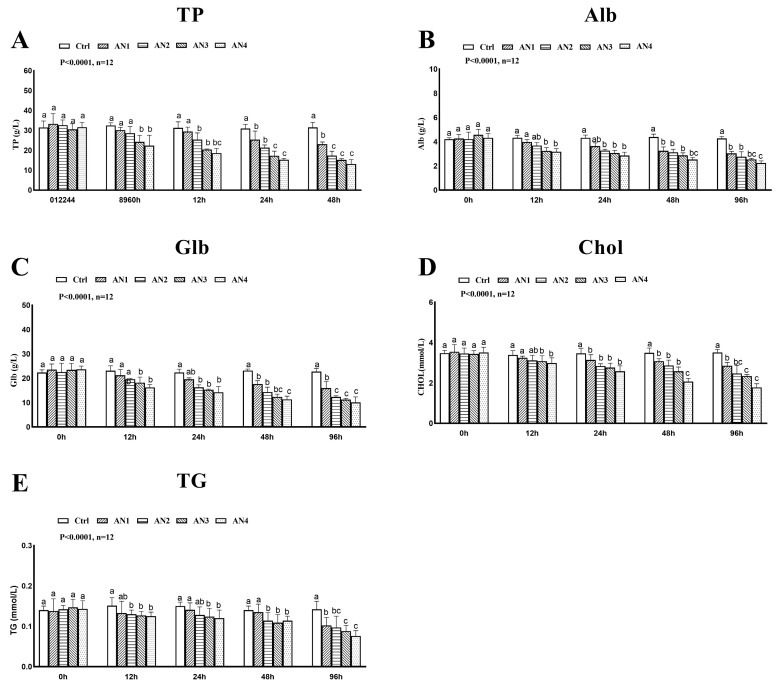
Changes of plasma biochemistry of bighead carp exposed to ammonia. (**A**) Serum total protein (TP), (**B**) albumin (ALB), (**C**) globulin (GLB), (**D**) total cholesterol (CHOL), and (**E**) triglycerides (TG). Values are expressed as mean ± standard error (SE) from triplicate groups (*n* = 12). Bars with different letters (a, b, c, differ significantly from those of other groups (*p* < 0.05).

**Figure 3:**
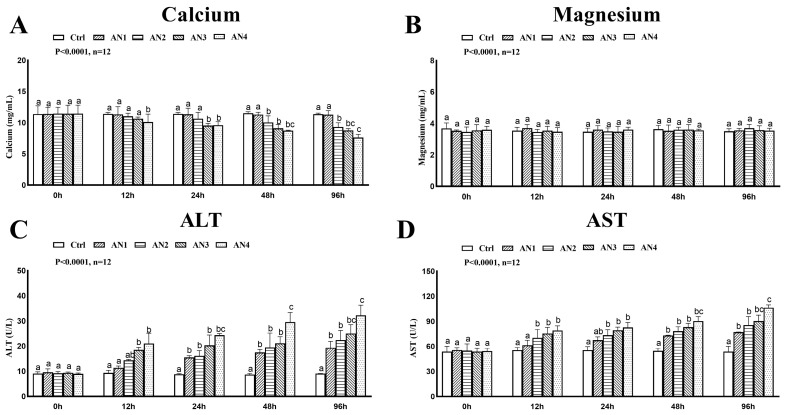
Changes of plasma biochemistry of bighead carp exposed to ammonia. (**A**) Calcium, (**B**) magnesium, (**C**) alanine aminotransferase (ALT), and (**D**) aspartate aminotransferase (AST). Values are expressed as mean ± standard error (SE) from triplicate groups (*n* = 12). Bars with different letters (a, b, c differ significantly from those of other groups (*p* < 0.05).

Compared with the control group, TP and Alb levels were significantly lower in the AN3 and AN4 groups at 12 h, 24 h, 48 h, and 96 h of ammonia exposure, Figure 2A,B. At 24 h, 48 h, and 96 h after ammonia exposure the GLB content of the AN2, AN3, and AN4 groups decreased significantly, Figure 2C. The CHOL content of AN1, AN2, AN3, and AN4 groups was significantly reduced by ammonia exposure for 24 h, 48 h, and 96 h, Figure 2D. After 12 h of ammonia exposure, the TG content in the AN2, AN3, and AN4 groups were significantly reduced, Figure 2E. The content of plasma calcium was significantly decreased in the AN3 and AN4 group at 24 h, 48 h, and 96 h of ammonia exposure, Figure 3A. There was no significant change in plasma magnesium content after exposure to ammonia, Figure 3B. The activities of AST and ALT increased significantly after 24 h of ammonia stress, Figure 3C,D.

### 3.4. Effect of Ammonia Exposure on Nonspecific Immunity

**Figure 4:**
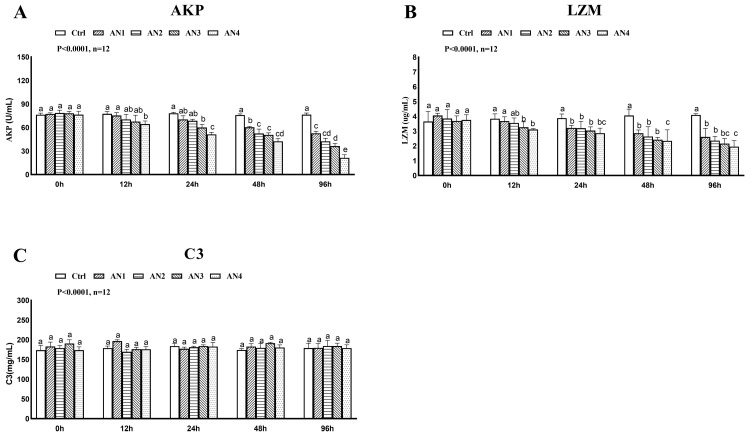
Changes of immune enzyme activity of bighead carp exposed to ammonia. (**A**) Alkaline phosphatase (AKP), (**B**) lysozyme (LZM), (**C**) complement 3 (C3). Values are expressed as mean ± standard error (SE) from triplicate groups (*n* = 12). Bars with different letters (a, b, c, d, e) differ significantly from those of other groups (*p* < 0.05).

The lysozyme activity was significantly decreased in the AN1, AN2, AN3, and AN4 groups at 24 h, 48 h, and 96 h of ammonia exposure, Figure 4B. After 48 and 96 h of ammonia exposure, AKP activity of the AN3 and AN4 groups decreased significantly, Figure 4A. Ammonia exposure had no significant effect on complement C3 content, see Figure 4C.

### 3.5. Effect of Nitrogen Stress on Stress Index of Bighead Carp

**Figure 5:**
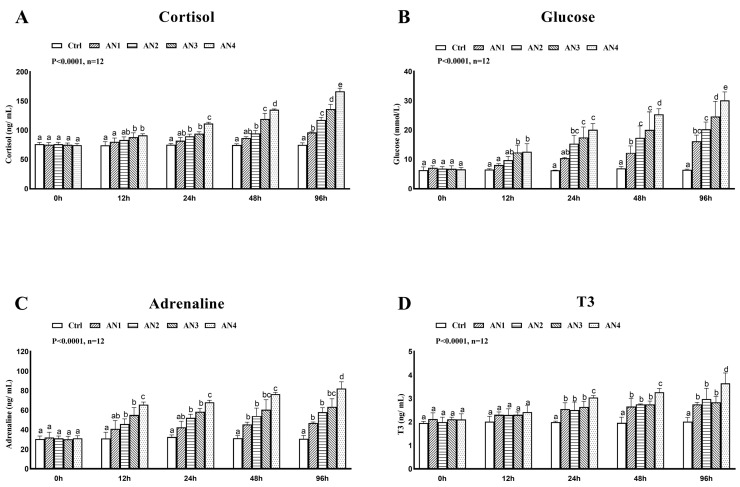
Changes of stress-related hormones in bighead carp exposed to ammonia. (**A**) Plasma cortisol, (**B**) glucose (Glu), (**C**) adrenaline, and (**D**) 3′-triiodothyronine (T3). Values are expressed as mean ± standard error (SE) from triplicate groups (*n* = 12). Bars with different letters (a, b, c, d, e) differ significantly from those of other groups (*p* < 0.05).

**Figure 6:**
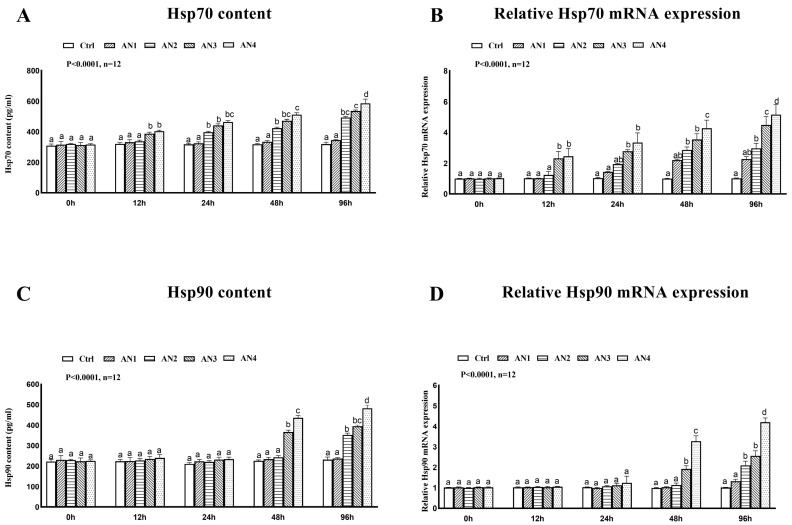
Changes of heat shock protein content and gene expression of bighead carp exposed to ammonia. (**A**) Heat shock protein 70 (HSP70), (**B**) relative *Hsp70* mRNA expression, (**C**) heat shock protein 90 (HSP90), and (**D**) relative *Hsp90* mRNA expression. Values are expressed as mean ± standard error (SE) from triplicate groups (*n* = 12). Bars with different letters (a, b, c, d) differ significantly from those of other groups (*p* < 0.05).

The content of COR, GLU, T3 and epinephrine in serum showed an increasing trend with the increase in stress time. The levels of cortisol, glucose and epinephrine in the AN2, AN3, and AN4 groups increased significantly at 12 h, 24 h, 48 h, and 96 h under ammonia exposure, Figure 5. The contents of T3 were significantly increased in the AN1, AN2, AN3 and AN4 groups at 24 h, 48 h, and 96 h under ammonia exposure, as shown in Figure 5D. The content of HSP70 protein in liver of the AN2, AN3, and AN4 groups exposed to ammonia at 24 h, 48 h, and 96 h increased significantly, Figure 6A. At 48 and 96 h after ammonia exposure, the content of HSP90 protein in AN3 and AN4 groups increased significantly, Figure 6C. After exposure to ammonia for 12 h and 24 h, there was no significant change in HSP90 protein content in each experimental group. There was no significant change in HSP70 and HSP90 protein content in AN1 in each time period, Figure 6.

### 3.6. Antioxidant Responses

**Figure 7:**
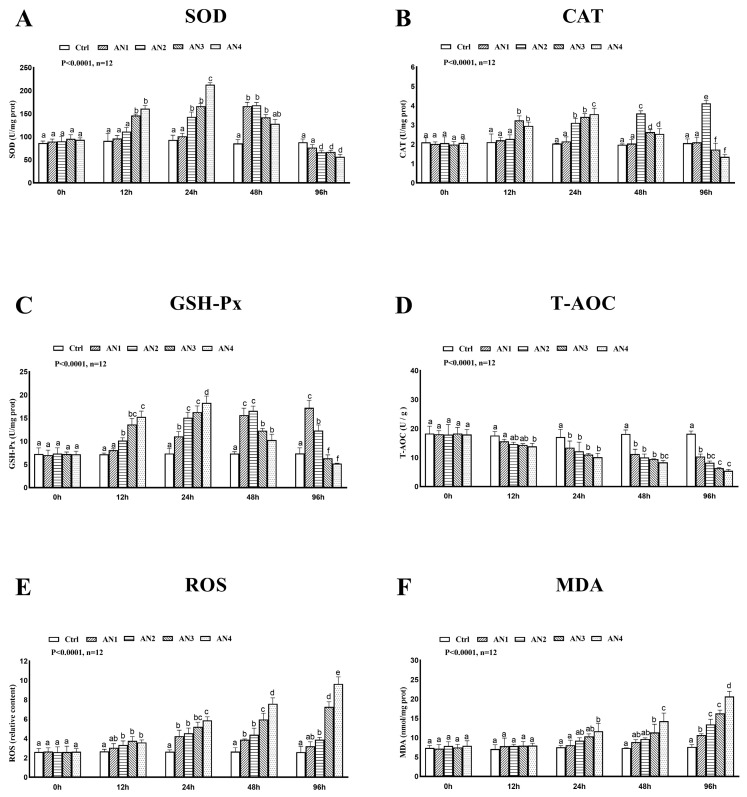
Changes of antioxidant enzyme activity of bighead carp exposed to ammonia. (**A**) Superoxide dismutase (SOD), (**B**) catalase (CAT), (**C**) glutathione peroxidase (GSH-Px), (**D**) total antioxidant capacity (T-AOC.), (**E**) reactive oxygen species (ROS), and (**F**) malondialdehyde (MDA). Values are expressed as mean ± standard error (SE) from triplicate groups (*n* = 12). Bars with different letters (a, b, c, d, e, f) differ significantly from those of other groups (*p* < 0.05).

At 24 h, 48 h, and 96 h after exposure to ammonia, T-AOC in the AN1, AN2, AN3, and AN4 groups decreased significantly, Figure 7D. ROS was significantly increased in the AN2, AN3 and AN4 groups at 12 h, 24 h, 48 h, and 96 h of ammonia exposure, Figure 7E. In the AN1 group, SOD activity was significantly increased in the AN1 group at 48 h of ammonia exposure, and decreased to no significant difference with the control group at 96 h of ammonia exposure. In the AN2 group, SOD activity was significantly increased at 24 h and 48 h and significantly decreased at 96 h. SOD activity in the AN3 group increased significantly at 12 h, 24 h, and 48 h, and decreased significantly at 96 h. The activity of SOD in the AN4 groups increased significantly at 12 h and 24 h, and decreased significantly at 96 h, Figure 7A. The activity of CAT in the AN3 and AN4 groups increased significantly after exposure to ammonia for 12 h, 24 h and decreased significantly at 96 h, Figure 7B. The activity of GPx in the AN2, AN3, and AN4 groups increased significantly after exposure to ammonia for 12 h, 24 h, and 48 h. The activity of GPx in the AN3 and AN4 groups was significantly lower at 96 h of ammonia exposure, as shown in Figure 7C. The content of MDA was significantly increased in the AN4 groups at 24 h, 48 h, and 96 h of ammonia exposure. The content of MDA in the AN3 groups was significantly increased at 48 h and 96 h of ammonia exposure, Figure 7F.

### 3.7. Effect of Ammonia Exposure on Cytokines

**Figure 8:**
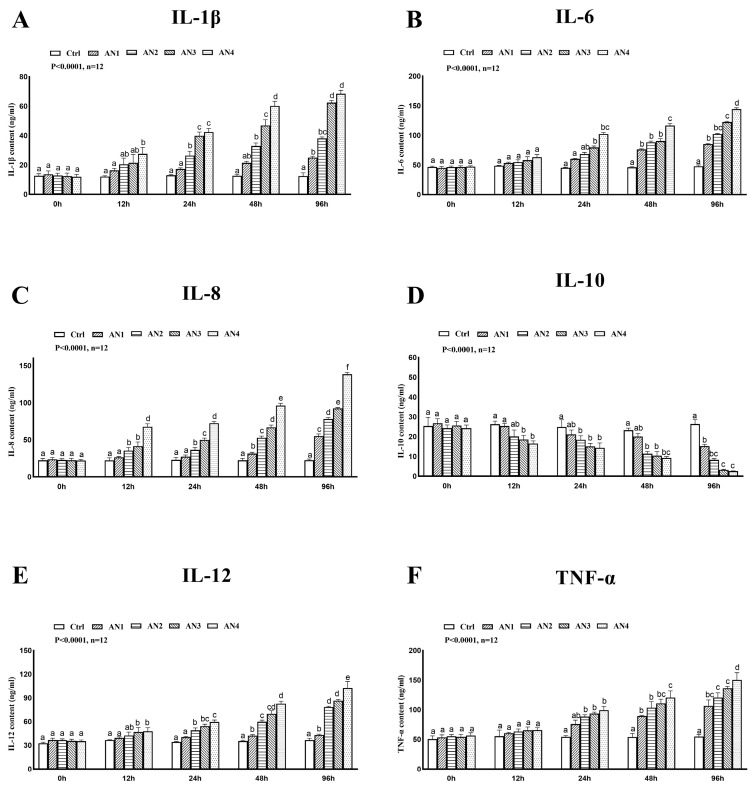
Changes of cytokine activity of bighead carp exposed to ammonia. (**A**) Interleukin-1β (IL-1β), (**B**) Interleukin-6 (IL-6), (**C**) Interleukin-8 (IL-8), (**D**) Interleukin-10 (IL-10), (**E**) Interleukin-12 (IL-12), (**F**) Tumor necrosis factor-α (TNF-α). Values are expressed as mean ± standard error (SE) from triplicate groups (n = 12). Bars with different letters (a, b, c, d, e, f) differ significantly from those of other groups (*p* < 0.05).

The changes in IL-1β, IL-6, IL-8, IL-12, TNF-α, and IL-10 cytokine levels after ammonia exposure are shown in Figure 8. In the present study, IL-1β, IL-6, IL-8, IL-12, and TNF-α activities were significantly increased in the AN3 and AN4 groups at 24 h, 48 h, and 96 h of ammonia exposure. The activity of IL-10 was significantly decreased in the AN3 and AN4 groups at 12 h, 24 h, 48 h, and 96 h of ammonia nitrogen exposure.

### 3.8. Effect of Ammonia Exposure on Gene Transcription

The expression of *HSP90* and *HSP70* mRNA genes under ammonia stress is shown in Figure 6. After exposure to ammonia for 12 h, 24 h, 48 h, and 96 h, the mRNA transcription level of *HSP70* in the liver of the AN3 and AN4 groups increased significantly. The mRNA expression of the *HSP70* gene of AN1 had no significant change in each time period, Figure 6B. The mRNA expression of the *HSP90* gene in the AN3 and AN4 groups increased significantly after 48 h and 96 h of ammonia exposure, but AN1 had no significant change at each time point. After exposure to ammonia for 12 h and 24 h, there was no significant change in the *HSP90* gene mRNA expression in each experimental group. The mRNA expression of the *HSP90* gene in AN1 had no significant change in each time period, Figure 6D.

## 4. Discussion

Excessive ammonia nitrogen in water will weaken the growth performance and health of aquatic animals. Ammonia exposure leads to an increase in the ammonia content in fish tissues, which leads to ammonia poisoning [22]. In this study, the 96 h LC50 concentration of NH_3_ for bighead carp was 0.854 mg/L, and the average 96 h LC50 concentration of NH_3_ for 32 common freshwater fish was 2.79 mg/L (NTIS), which was much higher than 0.854 mg/L in this experiment, which indicated that bighead carp was more sensitive to NH_3_ in water than most freshwater fish. Therefore, in the process of bighead carp breeding, the content of NH_3_ in water should be strictly controlled.

The changes in blood physiological indexes and hematological parameters of fish are closely related to metabolism, so they are often used to evaluate the health status of fish and their adaptation to the environment. The hematocrit, hemoglobin level, and red blood cells of hybrid groupers decreased significantly when exposed to ammonia [23]. The hemoglobin of crucian carp decreased significantly after being exposed to high concentration of ammonia [24]. Ammonia exposure significantly reduced the hemoglobin and hematocrit levels of Anoplopoma fimbria [25]. These studies suggest that ammonia exposure leads to tissue hypoxia, which damages hematopoietic cells and leads to inhibition and failure of hematopoietic potential. In our study, Hb content, Ht, and blood RBC count decreased significantly when exposed to high-concentration ammonia. Ammonia has high affinity and combines with hemoglobin to replace oxygen, which hinders the transfer of oxygen from gills to blood, causing hemolysis and further resulting in anemia and hemodilution [26,27]. The histological in bighead carp gills clearly showed a response to the ammonia concentrations. In the high concentration of ammonia, the secondary lamellae were severely disrupted, with severe edema underlying the lamellar epithelium and modest epithelial thickening [19]. At the same time, the increase in free radicals exposed to ammonia may attack red blood cells, leading to their destruction [28]. Ammonia toxicity leads to inhibition of hematopoiesis by disrupting erythropoietic sites. Therefore, the occurrence of anemia symptoms may be attributed to hypoxia, red blood cell destruction, and hematopoietic tissue damage under ammonia exposure. The decrease of white blood cells in fish may be related to nonspecific immunosuppression [29,30].

The increase in serum TP and ALB indicates the enhancement of the innate immunity of an organism [30]. In this experiment, with the prolongation of stress time, the serum TP and ALB contents of bighead carp decreased significantly, which indicated that ammonia stress had a significant effect on protein metabolism and reduced the nonspecific immunity of bighead carp. These results are consistent with those of golden pompano and yellow catfish when exposed to ammonia [13,31]. Ammonia exposure induces oxidative stress, and the resulting oxidative damage may be an important pathway for organ damage. Ammonia stress damages the kidney of fish, resulting in a large loss of blood protein excreted by the kidney, which may be the main reason for the decrease in serum protein. The contents of CHO and TG reflect the status of lipid absorption and fat metabolism [32]. With the prolongation of stress, the contents of CHOL and TG in the serum of bighead carp decreased significantly, which indicated that nitrogen stress affected the lipid metabolism and transport function of bighead carp.

As transaminases in the liver, ALT and AST activities are low and relatively stable under normal serum conditions. When hepatocytes are damaged, the synthesis of aminotransferases in the liver increase and are released into the circulatory system, resulting in an increase in serum transaminase activity. Therefore, serum ALT and AST activities can be used as indicators to evaluate the degree of liver damage in fish [33]. Many studies show that pollutants can significantly increase the ALT and AST activities of fish [34,35]. The plasma ALT and AST activities of redfin snapper [36] and blunt snout bream [12] significantly increased under ammonia exposure. Our findings were consistent with previous studies. In this experiment, it was found that both AST and ALT activities in serum increased significantly with increasing duration of ammonia stress, indicating that ammonia stress is capable of causing damage to the liver of bighead carp. Ammonia exposure also affects intracellular Ca^2+^ concentration, leading to the production of free radicals, which in turn leads to DNA damage, enzyme inactivation, and lipid peroxidation, and ultimately causes various physiological pathologies in the organism [7].

Non-ionic ammonia accumulates in fish tissues, which leads to the release of a large amount of reactive oxygen species, and the balance between reactive oxygen species production and antioxidant defense is damaged, which leads to oxidative stress and body dysfunction [37]. Oxidative stress is an important factor leading to the death of fish from ammonia poisoning. The antioxidant system can quickly remove ROS and plays a key role in protecting fish from ROS toxicity. T-AOC is a comprehensive index to measure the function of the antioxidant system, which is closely related to the health of fish. MDA is an important marker of lipid peroxidation, which reflects the oxidative damage of cells. The dynamic changes in MDA and T-AOC levels can fully reflect the oxidative stress status of the organism [38]. Many studies have demonstrated that antioxidant enzyme activity can be induced at low concentrations and impaired at higher concentrations of pollutants. The activity of antioxidant enzymes increased in a low concentration of pollutants but decreased in a high concentration of pollutants [4,39]. The gene expression of antioxidant enzymes in hybrid grouper [23] increased under low-concentration ammonia stress, and the ROS content increased sharply with the prolongation of stress time under high-concentration ammonia stress, while the antioxidant enzyme activity was significantly lower than that in the control group, suggesting that the antioxidant system was damaged. These studies suggest that the increase in antioxidant enzymes helps to scavenge the induced reactive oxygen species (ROS) and protects cells from oxidative damage in the early stages of ammonia exposure. However, when the antioxidant system can no longer remove higher ROS levels, it causes oxidative stress in the body. In this study, ammonia exposure led to a decrease in T-AOC and an increase in ROS and MDA content. The activities of SOD, CAT, and GPx first increased and then decreased. The ROS and MDA contents did not change significantly after 12 h of ammonia stress, which indicated that antioxidant enzymes could effectively remove the ROS produced in the metabolic process and prevent membrane lipid peroxidation and other damage. However, the scavenging of ROS by antioxidant enzymes was limited. With the increase in ammonia concentration and exposure time, the excessive production of ROS accelerated the depletion of antioxidant enzymes, which then damaged cell membranes and formed lipid peroxides, and MDA attacked the antioxidant defense system, leading to a decrease in antioxidant enzyme activity of bighead carp. Ammonia stress reduced the total antioxidant capacity of bighead carp. Enzyme activity is usually related to the expression of its coding gene at the transcriptional level. In our research, when exposed to different ammonia concentrations, the expression trend of antioxidant-enzyme related genes of bighead carp was consistent with that of antioxidant enzymes.

The innate immunity of fish is considered the first line of defense against external pathogens. LZM, AKP, and C3 are indexes used to define the immune performance of the body [40]. Lysozyme is widely present in the organism and its transcript level or activity is an important indicator of innate immunity in fish. In the present study, the activity of LZM and AKP of bighead carp decreased after ammonia exposure, which resulted in immunosuppression. The complement of fish can crack external cells, among which complement C3 plays a central role in the complement cascade reaction. In this study, ammonia exposure did not cause significant changes in C3 content. Similarly, there was no significant change in C3 content in the serum of blunt snout bream [12] or grass carp [6] under ammonia stress. The toxicity of ammonia reduced the immunity of aquatic organisms [41]. Ammonia stress caused a significant decrease in LZM activity and the immune function of blunt snout bream [12] and yellow catfish [4]. The effect of ammonia on fish immunity in intensive culture has become a hot issue.

When aquatic animals face adverse environmental stress, they need a lot of energy to maintain their internal balance. Adrenal tissues produce a lot of cortisol to promote the decomposition of hepatic glycogen and elevated blood glucose to help fish cope with various stresses, a process that is thought to be a detoxification mechanism for fish under stressful conditions [42]. Stress is a state of non-specific and physiological tension induced when animals are confronted with adverse environmental stimuli. The hypothalamus–pituitary–thyroid axis (HPT plays an important role in the regulation of the neuroendocrine system. The hypothalamus promotes TSH secretion and the thyroid gland is acted on by TSH, which secretes thyroxine and triiodothyronine (T3), which are released into blood and act on target cells, regulating the metabolism of sugar, protein, fat, water, and salt [43]. Cortisol is an important stress hormone secreted by the hypothalamus–pituitary–interrenal tissue axis (HPI) in fish after external stimuli. Cortisol usually rises rapidly under acute stress to enhance non-specific immunity in fish, regulate nutrition and physiological metabolism, and help the organism to counteract environmental stress. Therefore, elevated blood cortisol levels are seen as a sensitive signal of stress response in fish. Cortisol and blood glucose play an important role in the stress response and energy metabolism [44,45]. Plasma cortisol and blood glucose levels were elevated in rainbow trout [45] and common carp [30] under acute ammonia stress. In this experiment, serum T3, GLU, and cortisol levels of AN2, AN3, and AN4 groups were significantly elevated at 24 h of stress. This indicates that ammonia affects the hypothalamic–pituitary–thyroid axis in the neuroendocrine regulation of bighead carp. The serum T3 level was significantly increased by the stress, which affects its regulation of body sugar, protein, lipid, and water–salt metabolism, and regulates through the hypothalamic–pituitary–interrenal tissue axis cortisol, which promotes hepatic glycogenolysis and elevates blood glucose. Under ammonia stress, bighead carp responds quickly, which improves the ability of stress and nonspecific immunity to resist environmental stress.

Heat shock protein can prevent the aggregation of protein and help protein fold so as to maintain the stability of cells under stress and protect organisms from oxidative stress [46,47]. When exposed to environmental stress, the body synthesizes HSPs by activating heat shock genes. The increase in HSP70 and HSP90 levels may reflect the protective response of the body to environmental stress. HSP70 and HSP90 are often used as stress detection indicators [48,49]. In this study, it was found that the mRNA expression of the *HSP70* gene in the AN3 and AN4 groups showed a significant upward trend after 12 h, which indicated that the *HSP70* gene could respond quickly to ammonia nitrogen stress. Under ammonia stress, the mRNA expression of the *HSP70* gene in yellow catfish gills reached the highest value at 3 h [4]. This self-protection mechanism can largely reduce the damage caused by environmental stress to tissues in a short period of time. Compared with *HSP70*, the *HSP90* gene was not sensitive to environmental stress, and only when the stress was severe did it induce the transcription of the *HSP90* gene. This phenomenon was further confirmed by the present study, where the mRNA expression of the *HSP90* gene in the AN3 and AN4 groups showed a significant increase after 48 h. The mRNA expression of the *HSP90* gene in the liver and gill of yellow catfish in the high-concentration group showed significant upregulation only after 24 h of stress onset. The increase in *HSP70* and *HSP90* mRNA transcription level can help the balance of protein in the body, play a role in protecting cells and the immune system, and is the protective response of the body to ammonia stress.

There are multiple protective mechanisms against cell stress, but stress beyond the ability of cells to cope will lead to the interruption of cell signal transmission, DNA damage, tissue damage, and apoptosis. Inflammation is a normal defense response to tissue damage [50]. External stress can induce the maturation of inflammatory cytokines and participate in the regulation of the inflammatory response. The activity of inflammatory cytokines is an important index of animal immunity and anti-inflammatory response. IL-1β and TNF-α are regarded as important pro-inflammatory cytokines, and they participate in the inflammatory reaction as intermediate factors of the immune response when they are damaged by the external environment [51]. The exposure of ammonia nitrogen significantly increased the expression of IL-1β in common carp [29], blunt snout bream [12], and yellow catfish [4], and the acute stress of ammonia nitrogen increased the expression of TNF-α in common carp [30] and pufferfish [7], indicating that the body had an inflammatory reaction. In our study, with the increase in ammonia concentration and exposure time, the mRNA expressions of TNF-α, IL-6, IL-12, and IL-1β were significantly enhanced, and the expression of anti-inflammatory factor IL-10 was inhibited, which are results similar to those of previous studies. The exposure of ammonia interferes with the immune response of fish and causes inflammation.

## 5. Conclusions

Ammonia exposure caused changes in blood cell parameters and plasma components of bighead carp; induced increases in intracellular reactive oxygen species (ROS) content and MDA accumulation; resulted in decreases in antioxidant enzyme activity, serum protein, and lysozyme activity; and caused the up-regulation of inflammatory cytokines (TNF-α, IL-1β, IL-6 and IL-12). Furthermore, nitrogen exposure led to a rapid increase in stress indexes such as cortisol, blood sugar, adrenaline, and T3, and increased expressions of HSP70 and HSP90. Ammonia exposure caused oxidative stress, immunosuppression, inflammation, and the stress reaction of bighead carp. As far as we know, this study is the first detailed study on oxidation, immunity, inflammation, and the stress response of bighead carp exposed to ammonia. Our results will be helpful in understanding the mechanism of ammonia stress on the aquatic toxicology of bighead carp.

## Figures and Tables

**Table 1:** Water condition during the experiments.

	Ctrl	AN1	AN2	AN3	AN4	*p* Value
Temperature (°C)	22.62 ± 0.22	23.13 ± 0.32	22.55 ± 0.15	23.20 ± 0.16	23.14 ± 0.20	0.055
Dissolved oxygen (mg/L)	6.03 ± 0.11	6.11 ± 0.34	6.68 ± 0.36	6.29 ± 0.18	6.55 ± 0.08	0.062
pH	7.49 ± 0.14	7.52 ± 0.22	7.37 ± 0.05	7.49 ± 0.24	7.64 ± 0.20	0.231
TAN (mg/L)	0.68 ± 0.11	3.95 ± 0.21	7.91 ± 0.33	11.87 ± 0.15	15.82 ± 0.35	0.002
NH_3_ (mg/L)	0.04 ±0.01	0.21 ± 0.02	0.38 ± 0.01	0.62 ± 0.05	0.90 ± 0.07	0.021
Nitrite (mg/L)	0.050 ± 0.01	0.055 ± 0.01	0.051 ± 0.02	0.052 ± 0.01	0.053 ± 0.03	0.012

TAN: total ammonia nitrogen, NH_3_: un-ionized ammonia.

**Table 2:** Primers used in qRT-PCR.

Gene	Accession Number	Forward	Reverse
HSP70	AF210640.1	GGCCTGGACAAAGGCAAATC	CAGATGAGTGTCTCCAGCGG
HSP90	L35586.1	TTGAGGAAGGCGAGAAAGC	ATCCTCCCAGTCATTGCTTC
β-actin	AF301605	TGGATCGGAGGTTCCATCCT	TGGTCCAGACTCGTCGTACT

**Table 3:** LC50 of bighead carp exposed to ammonia.

	TAN (mg/L)	NH_3_-N (mg/L)
24 h LC50	26.05	1.41
48 h LC50	21.01	1.13
72 h LC50	17.06	0.92
96 h LC50	15.82	0.85

## Data Availability

Data available on request due to restrictions privacy. The data presented in this study are available on request from the corresponding author. The data are not publicly available due to restrictions privacy.
